# Dr. Charles Jewett

**Published:** 1910-09

**Authors:** 


### OBITUARY

---

Dr. Charles Jewett, of Brooklyn, died of apoplexy at his home on Saturday, August 6, 1910, aged 70 years. He was the president of the Medical Society of the State of New York, at the time of his death having been elected at the annual meeting held in January, 1910. He was a member of the various local medical societies, the New York Academy of Medicine, and the American Medical Association. He took his doctorate degree from the New York College of Physicians and Surgeons in 1871. He had been president of all the local medical societies in his city and county. He was Professor of Obstetrics and Gynecology in the Long Island College Hospital, and was the first to perform symphyseotomy in America. He was an author of distinction, his textbook on obstetrics standing at the fore in testimony thereof, while other contributions to literature mark him as a man of thought and action.

